# Ectopic expression of *Lc* differentially regulated anthocyanin biosynthesis in the floral parts of tobacco (*Nicotiana tobacum* L.) plants

**DOI:** 10.1186/s40529-016-0138-6

**Published:** 2016-09-08

**Authors:** Zong-An Huang, Ting Zhao, Ning Wang, Shu-song Zheng

**Affiliations:** 1 State Key Laboratory of Plant Cell and Chromosome Engineering, Institute of Genetics and Developmental Biology, Chinese Academy of Sciences, West Beichen Road 1, Chaoyang District, Beijing, 100101 China; 2 Institute of Vegetable Sciences, Wenzhou Academy of Agricultural Sciences, Wenzhou Vocational College of Science and Technology, Wenzhou, 325014 China

**Keywords:** Anthocyanin-biosynthetic genes, Cyanidin, Flower colour, *Lc*, Tobacco (*Nicotiana tobacum* L.)

## Abstract

**Background:**

Anthocyanins are the conspicuous pigments of flowering plants and participate in several aspects of plant development and defense, such as seeds and pollens dispersal. *Leaf colour* (*Lc*) is the first basic/helix-loop-helix (bHLH) transcription factor controlling anthocyanin biosynthesis isolated from maize (*Zea mays* L.). Ectopic expression of maize *Lc* enhanced anthocyanin biosynthesis in many plants including tobacco (*Nicotiana tobacum* L.). However, the molecular regulatory mechanism of anthocyanin biosynthesis in the different floral parts of tobacco remains largely unknown. Therefore, the molecular and biochemical characterization of anthocyanin biosynthesis were investigated in the flowers of both wild type and *Lc*-transgenic tobacco plants.

**Results:**

At the reproductive stage, with respect to the different parts of the flowers in wild type *SR1*, the calyxes and the pistils were green, and the petals and the filaments showed light pink pigmentation; the *Lc*-transgenic tobacco exhibited light red in calyxes and crimson in petals and in filaments respectively. Correspondingly, the total anthocyanin contents (TAC) in calyxes, petals and filaments of *Lc*-transgenic plants were much higher than that of the counterparts in *SR1*. Though the TAC in anthers of *Lc*-transgenic plants was low, it was still significantly higher than that of *SR1*. *SR1* has almost the same TAC in the pistils as *Lc*-transgenic plants. Consistent with the intense phenotype and the increased TAC, *Lc* was weakly expressed in the calyxes and strongly expressed in petals and filaments of *Lc*-transgenic plants, while *Lc* was not detected in *SR1*. The expression level of *NtAN2* in petals was similar between SR1 and *Lc*-transgenic lines. In agreement with the expression profile of *Lc*, both early (*NtCHS*) and late anthocyanin-biosynthetic genes (*NtDFR*, *NtF3′H*, and *NtANS*) were coordinately up-regulated in the counterparts of flowers. HPLC analysis demonstrated that the cyanidin (Cya) deposition was mainly responsible for the intense pigmentation of *Lc*-transgenic tobacco.

**Conclusions:**

Ectopic expression of *Lc* greatly enhanced both early- and late- anthocyanin-biosynthetic gene expression, and therefore resulted in the Cya-based TAC increase in the calyxes, the filaments and the petals in tobacco plants.

## Background

Anthocyanins represent the most conspicuous class of flavonoids owing to their striking colours ranged from orange to red to blue. The striking colours not only facilitate the pollination and seed dispersal but also contribute to plant adaptation to environmental stresses, such as UV light stress (Campanella et al. 2014). The regulation of anthocyanin biosynthesis is extensively studied in higher plants. Anthocyanin biosynthesis is a branch of flavonoid pathway, which is usually categorized as either early flavonoid biosynthetic genes (EBGs) or late biosynthetic genes (LBGs) (Quattrocchio et al. 1993; Mol et al. 1998). The EBGs encode enzymes such as the chalcone synthase (CHS), chalcone isomerase (CHI), and flavanone 3-hydroxylase (F3H) (Quattrocchio et al. 1993). The LBGs lead to the production of the anthocyanins, involving the dihydroflavonol reductase (DFR), anthocyanidin synthase (ANS) and UDPG: flavonoid 3-*O*glucosyltranferase (UFGT) (Quattrocchio et al. 1993; Mol et al. 1998).

The control of flavonoid biosynthesis pathway is largely at the level of transcription of regulators and of the corresponding biosynthetic genes. Three types of transcription factor, i.e. R2R3 MYB, bHLH and WD40, have been reported for the regulation of anthocyanin biosynthesis at the transcript level (Xie et al. 2016). *Lc* was the first characterized *myc* bHLH transcription factor, which was involved in the regulation of anthocyanin biosynthesis. *Lc* controlled the chalcone synthase gene (*CHS*) and dihydroflavonol 4-reductase gene (*DFR*) in maize (Ludwig et al. 1989), and it also regulated the flavonoid pathway in both monocots and dicots. There have been an increasing number of reports on genetic engineering of anthocyanin biosynthesis pathway for floricultural and agricultural purposes.

Heterologous expression of *Lc* enhanced anthocyanin biosynthesis in tobacco, *Petunia* and *Caladium bicolour* at both vegetative stage and floral stage (Lloyd et al. 1992; Bradley et al. 1998; Bovy et al. 2002; Li et al. 2005; Albert et al. 2009). Moreover, expression of *Lc* in rice (*Oryza sativa* L.) resulted in red spikelet and caused sterility (Song et al. 2013). Recently, ectopic expression of *Lc* in cotton (*Gossypium hirsutum* L.) promoted its anthocyanin biosynthesis in cotton fiber and increased its tolerance to bollworm, while expression of *Lc* in sweet potato (*Ipomoea batatas* L.) enhanced anthocyanin and lignin biosynthesis (Fan et al. 2016; Wang et al. 2016). The effects of *Lc* in different plants might depend on the specificity of plant species and their growth conditions.

Tobacco is a commonly used heterologous system to investigate the gene functions in plants (Pattanaik et al. 2010). *Lc* was found to be coordinated with some transcription factors such as R2R3 MYB transcription factor to regulate anthocyanin biosynthesis (Franken et al. 1994). In tobacco, two types of transcription factors, bHLH transcription factor (NtAN1a, NtAN1b) and R2R3 MYB (NtAN2) were characterized and expressed predominantly in flowers and in juvenile leaves under low-temperature stress (Bai et al. 2011; Pattanaik et al. 2010; Huang et al. 2012). Though the change of flower colour of *Lc*-transgenic tobacco has been reported (Lloyd et al. 1992; Yang et al. 2007), the molecular mechanism underlying the enhanced pigmentation in different floral parts of *Lc*-transgenic tobacco plants remains unclear. Here we investigated the different impacts of heterologous expression of *Lc* upon anthocyanin biosynthesis in the calyxes, petals, filaments, stamens and pistils of tobacco flowers and identified the specific anthocyanidin responsible for intense pigmentation in *Lc*-transgenic tobacco plants.

## Methods

### Plant growth conditions and treatments

The production of *Lc*-transgenic plant lines were described by Huang et al. (2012). Seeds of wild type tobacco *SR1* (*Nicotiana tobacum* L.) and the T_2_ seeds of transgenic lines were surface-sterilized in 30 % household bleach with 0.01 % of Tween, and then washed with sterilized water for three times. The seeds were sown on the MS medium. After two true leaves emerged, the seedlings were transplanted into sand culture irrigated with Hoagland’s solution with the concentration of 20 μmol/L of Fe-Na_2_EDTA.

The T_2_ *Lc*-transgenic lines were cultivated in the soil pots in greenhouse and they were characterized carefully, especially for the flower organs. At the reproductive stage, the full expanded flowers prior to fertilization were collected for anthocyanin quantification and RNA gel assay (Nishihara et al. 2005). Different parts were from at least 5 intact flowers.

### Northern blot analysis

Total RNAs were extracted using Trizol (Invitrogen, USA) from different floral parts in *SR1* and *Lc*-transgenic lines. Fifteen micrograms of RNA was used for RNA gel assay. RNA hybridization and detection were performed according to the previous methods (Sambrook et al. 2001). Seven genes (*Lc, NtPAL*, *Nt4CL*, *NtCHS*, *NtDFR*, *NtANS*, and *NtGST*) were analyzed. The probes were amplified with the primers listed in Table 1.

**Table 1:** Primers used for amplification of probes in Northern blot assay

Gene	Forward primer (5′–3′)	Reverse primer (5′–3′)
*Lc*	GGATC ATGGCGTTTCAGCTTC	GGTAACCTCACCGCTTCCCTATAGCT
*PAL*	TCCTTTCACTTCGCTCCAAC	GGGAGAAAATTGAGGGGTTA
*4CL*	ACACAATCCGATGAGCATGA	CCCCAGACATGACAGTCCTT
*CHS*	TGAAAAATCGATGATTAAGAAGAGG	TCTGGAATTGGATCAGAACCTATAA
*DFR*	ACAACAAGAAGGTCAAGCATCTATT	CCAGTAATTAGTGAAAGTGCAGTGA
*ANS*	GTTTTCCCCGAGGACAAGTG	TTTCAAGGGTGTCCCCAATA
*GST*	AGCCAATGTTGGGAATGGTA	TTCCCGAAGGACATGTTAGG
*AN2*	CCTCATGATCAAAAGGAGAGC	AGAAGTGGCATTTCCTCATGC

### Multiplex RT-PCR assay

Taking the petals as samples, ten structural genes (*NtPAL*, *Nt4CL*, *NtC4H*, *NtCHS*, *NtCHI*, *NtF3H*, *NtF3'H*, *NtDFR*, *NtANS* and *NtFLS*) and two regulatory genes (*Lc* and *NtAN2*) were selected and analyzed with multiplex RT-PCR. The sequences of the primer pair and the multiplex RT-PCR procedure were described previously in Huang et al. (2012).

### Quantification of total anthocyanin content (TAC) and HPLC assay of cyanidin, delphinidin and pelargonidin

Different parts from at least 5 intact flowers were collected as samples. The total TAC assay was carried out as described in Huang et al. (2012). To investigate the components of the intensified pigmentations in petals of *Lc*-transgenic tobacco, the anthocyanin was extracted and analyzed from the pool with at least five petals from *SR1* and *Lc*-transgenic plants (Wang et al. 2006). The anthocyanin standards, cyanidin (Cya), delphinidin (Del) and pelargonidin (Pel) (Sigma) were dissolved in methanol to prepare the storage concentration at 0.1, 0.1 and 0.5 mg/mL, respectively. The HPLC procedure was carried out as described by Merken and Beecher (2000) with slight modifications. Briefly, the anthocyanin extracts were separated through a Zorbax Eclipse XDB-C18 column (Hewlett Packard) (250 × 4.6 mm, 5 μm) preceded by a guard column (12.5 × 4.6 mm) with the same stationary phase, and both columns were maintained at 30 °C. The flow rate was 1 mL/min. Each injection volume was 20 μL. Using a gradient of acetonitrile in 0.05 % (w/w) trifluoroacetic acid, the LC system Schimazu 10A vp was used and the consecutive assay from 200 to 600 nm. On the basis of the specific retention time, the Cya, Del and Pel were assayed with a photodiode array detector Merken and Beecher (2000).

## Results

### *Lc*-transgenic tobacco lines exhibited more intense pigmentation in floral parts

**Fig. 1:**
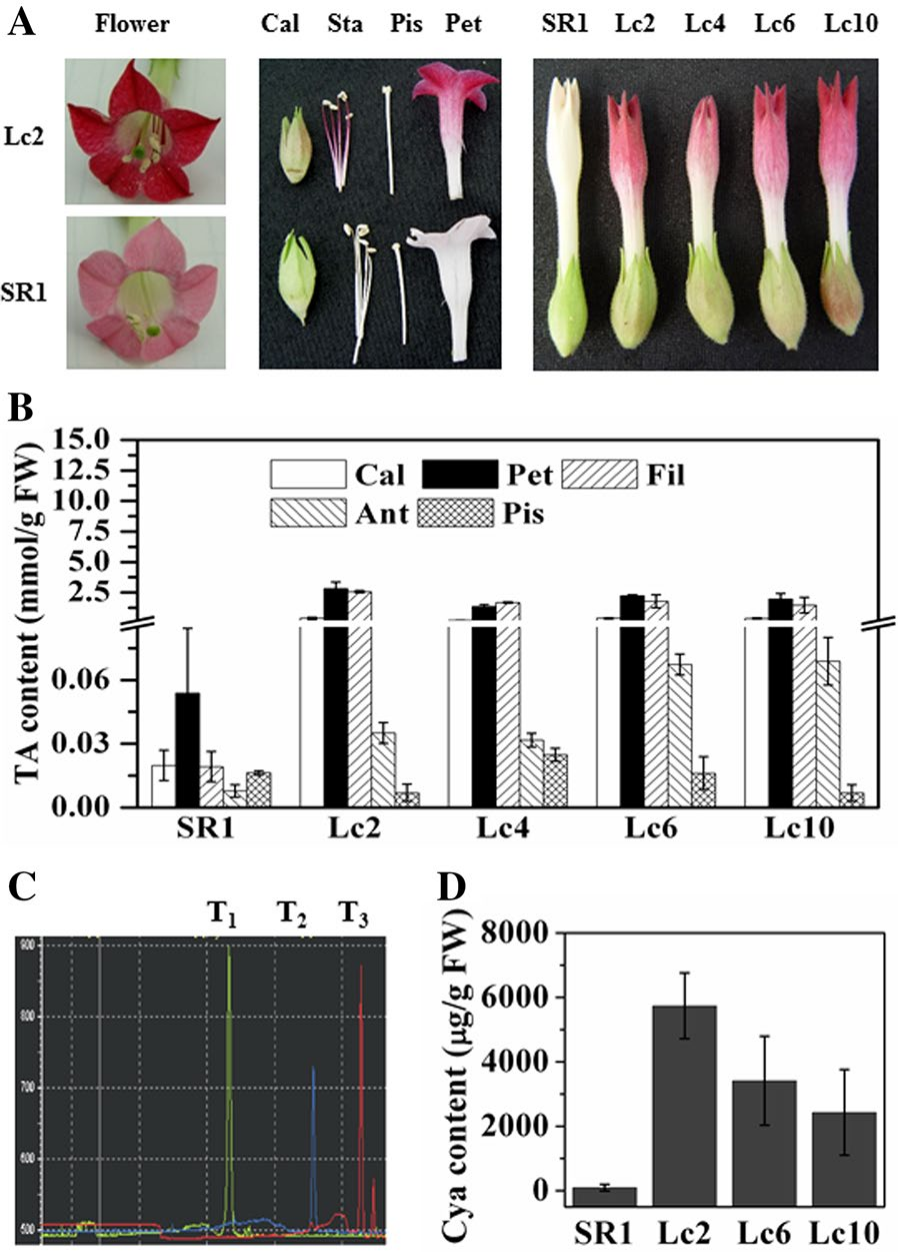
Phenotypic comparison of the intact flower and its five different parts (calyx: Cal; Petals: Pet; filaments: Fil; stamen: Sta; pistil: Pis) between *SR1* (WT) and four *Lc*-transgenic lines grown in the greenhouse (**A**); quantification of total anthocyanin content in the Cal, Pet, Fil, Sta and Pis of *SR1* and *Lc*-transgenic plants (**B**); In HPLC assay, the retention time of three standard anthocyanins (the cyanidin-Cya, *blue peak*; the delphinidin-Del, *green peak*; the pelargonidin-Pel, *red peak*) was 32.148, 37.960 and 41.213 min, respectively (**C**); HPLC assay of Cya concentrations in petals of *SR1* and *Lc*-transgenic lines (**D**)

More than 10 *Lc*-transgenic plants were cultivated and screened, among which, the lines *Lc2*, *Lc4*, *Lc6*, and *Lc10* were used for the following assay. For *SR1*, at the vegetative stage, the leaves and stems were green, while at the bloom stage, the calyxes were green, the petals and filaments were pale red, and the anthers as well as the stamens exhibited light green. For *Lc*-transgenic lines, green leaves and stems were similar to those of *SR1*, while the flowers appeared light red. The colour intensity was consistent with the TAC in the different floral organs. The TAC in young and mature leaves at the seedling stage as well as the anthers and the pistils at floral stage was very low, and the TAC was greatly increased in calyxes, in petals, and in filaments. Flowers of all *Lc*-transgenic lines displayed dark red (Fig. 1A). As compared to the counterparts in *SR1*, the TAC in calyxes, in petals, and in filaments of the *Lc*-transgenic lines was increased by 18, 51 and 132-folds, respectively. Though the TAC in anthers was low, it was still much higher than that of *SR1* (Fig. 1B).

### Cyanidin is responsible for the intense pigmentation in *Lc*-transgenic tobacco lines

To clarify the major component of anthocyanins responsible for intense pigmentation in *Lc*-transgenic flowers, three most common anthocyanins, Cya (cyanidin), Pel (pelargonidin), and Del (delphinidin) were further quantified with HPLC. The HPLC extraction and analysis system of the three anthocyanidins were established. The retention times of Cya, Del and Pel standard sample were 32.148, 37.960 and 41.213 min, respectively (Fig. 1C). Only Cya was identified in *Lc*-transgenic tobacco petals. Moreover, the Cya concentrations of three *Lc*-transgenic lines were at least 38-fold higher than that of *SR1* (Fig. 1D), which demonstrated that the increased cyanidin was responsible for the intense pigmentation in the petals.

### Boosted expression of key anthocyanin-biosynthetic genes in *Lc*-transgenic lines

**Fig. 2:**
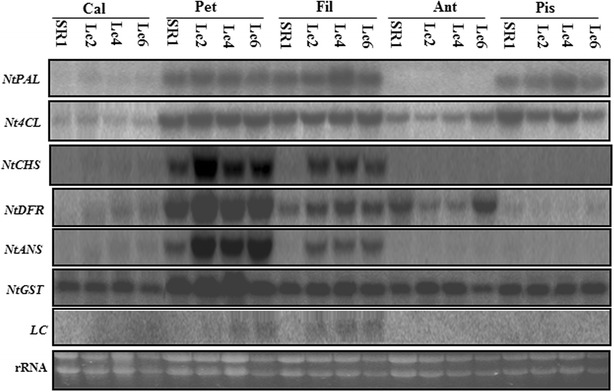
Northern blot analysis of anthocyanin biosynthetic genes. The genes *NtPAL*, *Nt4CL*, *NtCHS*, *NtDFR*, *NtANS* and *NtGST* encode phenylalanine ammonia-lyase, 4-coumaroyl-CoA ligase, chalcone synthase, dihydroflavonol 4-reductase, anthocyanidin synthase and glutathione S-transferase, respectively. *Lc* is the maize anthocyanin regulatory gene

Intense pigmentation was detected in the floral parts, especially in petals and in filaments of *Lc*-transgenic plants, compared to *SR1* plants, which suggested that ectopic expression of *Lc* was responsible for the anthocyanin increases. Northern blot displayed that *Lc* expressed comparatively weak in calyxes, strong in petals and filaments (Fig. 2), which agreed with the differential TAC increases in calyx, petals and filaments in *Lc*-transgenic lines (Fig. 1).

To elucidate how ectopic expression of *Lc* enhanced anthocyanin biosynthesis in the different parts of *Lc*-transgenic flowers, six key anthocyanin biosynthetic genes (*NtPAL*, *Nt4CL*, *NtCHS*, *NtDFR*, *NtANS* and *NtGST*) were analyzed with Northern blot. *NtPAL* was highly expressed in the petals, filaments and pistils and its expression pattern were similar between *SR1* and *Lc*-transgenic lines. *Nt4CL* expressed weakly in the calyxes and anthers, strongly in the petals, filaments and pistils in both *SR1* and *Lc*-transgenic lines. *NtCHS* expressed exclusively in petals of *SR1*, while it was greatly upregulated in petals and filaments in *Lc*-transgenic lines. *NtDFR* expressed highly in the petals, filaments and anthers, low in the calyx, and absent in the pistils of *SR1*, while it expressed significantly higher in the petals and filaments of *Lc*-transgenic lines. Similar expression profile was detected for both *NtANS* and *NtCHS*, with an exclusive expression in petals in *SR1*, and a significantly upregulated expression in the petals and filaments in *Lc*-transgenic lines. *NtGST* was ubiquitously expressed in the five floral parts of *SR1* and *Lc*-transgenic lines (Fig. 2).

### The anthocyanin biosynthesis pathway was enhanced in petals of *Lc*-transgenic plants

**Fig. 3:**
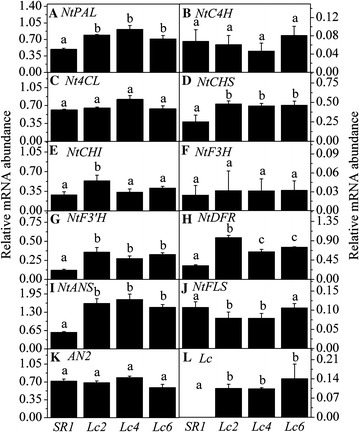
Multiplex RT-PCR expression analysis of the twelve flavonoid-related biosynthetic genes in petals of SR1 and three Lc-transgenic tobacco lines. **A** *NtPAL*, phenylalanine ammonia-lyase; **B** *NtC4H*, cinnamate 4-hydroxylase; **C** *Nt4CL*, 4-coumaroyl-CoA ligase; **D** *NtCHS*, chalcone synthase; **E** *NtCHI*, chalcone isomerase; **F** *NtF3H*, Flavanone 3-hydroxylase; **G** *NtF3´H*, flavonoid 3´-hydroxylase; **H** *NtDFR*, dihydroflavonol 4-reductase; **I** *NtANS*, anthocyanidin synthase; **J** *NtFLS*, flavonol synthase; **K** *NtAN2*, R2R3-MYB transcription factor, anthocyanin regulatory genes in tobacco (Nicotiana tobacum L.); **L** *Lc*, leaf colour gene in maize (Zea mays L.), anthocyanin regulatory gene

To further discriminate the molecular mechanism of the TAC increase in the *Lc*-transgenic lines, we analyzed the expression of genes involved in the anthocyanin biosynthetic pathway in the petals with multiplex RT-PCR. *Lc* was only detected in the petals of three *Lc*-transgenic lines (Fig. 3L), the expression of *NtPAL, NtCHS*, *NtCHI*, *NtF3′H*, *NtDFR*, *NtANS* increased by 47, 47, 70, 170, 139 and 124 % in *Lc*-transgenic line as compared to those in *SR1* (Fig. 3A, D, E, G–I). *Nt4CL* and *NtF3H* expressed similarly between *SR1* and *Lc*-transgenic lines (Fig. 3C, F). Additionally, the flavonol synthase gene *NtFLS* expression was much lower in the two *Lc*-transgenic lines than that in *SR1* (Fig. 3J).

For the anthocyanin regulatory gene, *Lc* was only detected in *Lc*-transgenic lines (Fig. 3L) not in *SR1*. The expression of *NtAN2,* the partner of *Lc*-like bHLH proteins in regulating the anthocyanin biosynthesis pathway was not changed in the *Lc*-transgenic lines (Fig. 3K), which suggested that ectopic expression of *Lc* rather than *NtAN2* is responsible for the up-regulation of anthocyanin biosynthesis genes as well as the anthocyanin accumulation in the *Lc*-transgenic lines.

Taken together, the four key genes *NtCHS*, *NtF3’H*, *NtDFR* and *NtANS* were greatly upregulated in petals and filaments of *Lc*-transgenic lines, which resulted in enhanced anthocyanin biosynthesis and more intense pigmented calyx, petals and filaments in the *Lc*-transgenic lines.

## Discussion

Great efforts have been made to modify the flower colour by manipulating the structural genes (such as *DFR, CHS,* and *ANS*) or their regulatory genes (such as *Lc*, *Pl*) (Nishihara and Nakatsuka 2011; Grotewold 2006; Han et al. 2009). The first introduction of maize *A1* gene encoding DFR into petunia enhanced the pelargonidin synthesis and resulted in brick red pigment in flower (Meyer et al. 1987). Recently, transcription factors controlling the genes involved in anthocyanin biosynthesis have been characterized not only in model plants, but also in floricultural plants (e.g. petunia, snapdragon) and food crops. The strategy by manipulating the transcription factors can modify multiple anthocyanin biosynthetic genes effectively. Heterologous expression of *AtPAR* in *Taraxacum brevicorniculatum* resulted in a red/purple vegetative tissue (Qiu et al. 2014). Ectopic expression of maize *Lc* greatly boosted the production of anthocyanin in cotton and in sweet potato (Fan et al. 2016; Wang et al. 2016). In this study, heterologous expression maize *Lc* in tobacco exhibited more intense pigmentation in the floral tissues (Fig. 1A) similar to the previous report by Lloyd et al. (1992) and Yang et al. (2007). The specific expression of *Lc* in the calyx, petals and filaments increased the transcript abundance of anthocyanin biosynthetic genes (Figs. 2, 3), which resulted in the higher anthocyanin content, and led to the more intense pigmentation in floral parts (Fig. 1).

The more intense pigmentation in floral parts of *Lc*-transgenic tobacco plants (Fig. 1A) should be due to systematic effects of ectopic expression of *Lc.* Ectopic expression of *Lc* in petals enhanced both early flavonoid-biosynthetic (*NtCHS* and *NtF3′H*) and late flavonoid biosynthetic genes (*NtDFR*, *NtANS*) (Figs. 2, 3), which was different in petunia or rice. In *Lc*-transgenic petunia, *CHS*, *CHI*, *F3H* was weakly upregulated, while *DFR*, *F3′H*, *F3′5′H*, *ANS* and *UFGT* were highly increased; *PAL*, *3RT*, and *C4H* were not affected (Bradley et al. 1998). In rice, *CHS*-like gene was increased in *Lc*-transgenic rice and might lead to sterility (Song et al. 2013). All these studies suggested that ectopic expression of *Lc* in different species regulated the common targets (such as *CHS* and *DFR* genes). However, the effects of ectopic *Lc* differed in the different plant species or even tissues.

Flavonoids shares a basic C_6_–C_3_–C_6_ skeleton structure in common consisting of two aromatic rings (A and B) and a heterocyclic ring C containing one oxygen atom (Halbwirth et al. 2010). The anthocyanins are one of the major classes of the flavonoids according to the oxidative status of the ring C. Delphinidin, pelargonidin and cyanidin differ in the different positions of hydroxyl of the B-rings. From a biochemical perspective, anthocyanin biosynthesis can be controlled by two processes, one is the number of hydroxyls of B-rings; the other is the substrate specificity of dihydroflavonol 4-reductase (DFR) (Meyer et al. 1987). For the former reaction, dihydrokaempferol (DHK) can be hydroxylated by flavonoid 3′-hydroxylase (F3′H) to produce dihydroquercetin (DHQ) or be changed to dihydromyricetin (DHM) by flavonoid 3′, 5′-hydroxylase (F3′5′H), respectively (Holton and Cornish, 1995). For the latter reaction, DHK cannot be catalyzed by DFR efficiently in some plant species, such as in *petunia* and tobacco (Forkmann and Ruhnau 1987). In this study, the expression of *F3′H* in *Lc*-transgenic lines was significantly increased than that in *SR1* (Fig. 3), which might be partly responsible for the increase in Cya content rather than Del or Pel. Aharoni et al. (2001) found that ectopic expression of *FaMYB1*, a strawberry fruit ripening transcription factor containing the repressor domain, resulted in the reduced Cya content and other flavonoid content, which in turn confirmed that Cya is the major anthocyanin determines the petal colour in tobacco plants.

## Conclusion

Taken together, heterologous expression of maize *Lc* gene enhanced the anthocyanin biosynthesis in the calyx, petals and filaments by the upregulation of both early and late flavonoid biosynthetic genes. Consequently, the anthocyanin accumulated especially in the Cya content.

## Abbreviations

4CL: 4-coumaroyl-CoA ligase
ANS: anthocyanidin synthase
C4H: cinnamate 4-hydroxylase
CHI: chalcone isomerase
CHS: chalcone synthase
DFR: dihydroflavonol 4-reductase
FLS: flavonol synthase
GST: glutathione S-transferase
F3H: flavanone 3-hydroxylase
F3′H: flavanone 3′-hydroxylase
F3′5′H: flavanone 3′5′hydroxylase
HPLC: high performance liquid chromatography
PAL: phenylalanine ammonia-lyase
TAC: total anthocyanin content
